# Soil Microbial Diversity and Its Environmental Drivers in the Rhizosphere Profile of *Camellia reticulata*

**DOI:** 10.3390/microorganisms14040806

**Published:** 2026-04-01

**Authors:** Fu-Jun Yan, Chong Ma, Hong-Xing Xiao, Yu-Jia Zeng, Yuan-Yuan Huang, Zhi-Yu Zhang, Zhong-Lang Wang, Long-Qing Chen, Fang Geng

**Affiliations:** 1 College of Landscape Architecture and Horticulture, Yunnan Key Laboratory of Landscape Plant Resource Cultivation and Application, Southwest Forestry University, Kunming 650224, China; yfj940919436@163.com (F.-J.Y.); machong@swfu.edu.cn (C.M.); hongxing1222025@163.com (H.-X.X.); 18287788533@163.com (Y.-J.Z.); 13698707504@163.com (Y.-Y.H.); zhangzhiyu1111@swfu.edu.cn (Z.-Y.Z.); chenlq@swfu.edu.cn (L.-Q.C.); 2 Kunming Institute of Botany, Chinese Academy of Sciences, Kunming 650201, China; wang@mail.kib.ac.cn

**Keywords:** *C. reticulata*, soil microbial diversity, rhizosphere soil, environmental factors

## Abstract

To investigate the main drivers of rhizosphere soil microbial community structure and diversity in *Camellia reticulata*, this study collected rhizosphere soil samples from six regions at two soil depths (0–30 cm and 30–60 cm). Using high-throughput sequencing, we systematically analyzed the effects of soil environmental factors on microbial communities. The results showed that the dominant bacterial phyla were Proteobacteria, Acidobacteriota, Chloroflexi, Actinobacteriota, and Bacteroidota, while the dominant fungal phyla were Ascomycota, Basidiomycota, and Mortierellomycota. Alpha diversity of both bacterial and fungal communities was higher in surface soils (0–30 cm) than in deeper layers (30–60 cm), although the differences were not statistically significant (*p* > 0.05). Soil pH, potassium content (K), and catalase activity (S-CAT) were identified as the main environmental factors significantly correlated with microbial community structure. Network analysis identified Acidobacteriota and Ascomycota as highly connected taxa within microbial networks, suggesting their potential importance in maintaining network structure. This study reveals the vertical differentiation characteristics of rhizosphere microbial communities in *C. reticulata* and their responses to environmental factors, providing a theoretical basis for cultivation management and rhizosphere microecological regulation.

## 1. Introduction

*Camellia reticulata* is an evergreen tree or shrub belonging to the family Theaceae and the genus *Camellia*. As an important ornamental plant endemic to Yunnan, China, it is characterized by large flower size, diverse coloration, and varied floral forms, conferring high horticultural value [1,2]. In terms of natural distribution, *C. reticulata* exhibits pronounced regional specificity, being primarily concentrated in parts of southwestern China [3], with a cultivation history spanning over 1300 years [4]. Despite its significant ornamental and economic value, *C. reticulata* has relatively strict soil requirements, making its cultivation challenging. Therefore, how to overcome the difficulty in cultivating *C. reticulata* by improving the soil or cultivation substrate has always been a key concern for its growers and researchers and is of great significance to the development of the *C. reticulata* industry.

As a key medium for plant growth and development, soil not only supplies water and nutrients but also profoundly influences root architecture, microbial interactions, and physiological-metabolic processes [5]. Rhizosphere microorganisms are widely recognized as key mediators of plant–soil interactions, playing crucial roles in nutrient cycling, stress resistance maintenance, and microecological balance [6,7,8]. Due to the gradients in carbon availability, nutrient limitation, and oxygen content with increasing soil depth, microbial communities exhibit significant heterogeneity in their vertical distribution [9].

In *Camellia* plants, existing studies have confirmed that agricultural management practices and host characteristics are key drivers shaping rhizosphere microecology. Gu et al. [10] demonstrated that fertilizer type not only significantly alters the diversity of soil microbial communities in *C. sinensis* but also reshapes their microbial network structure. Tang et al. [11] further revealed that although the interaction between cultivar characteristics and management practices leads to specific differentiation in the rhizosphere community structure of *C. oleifera*, core community similarity is maintained across different habitats. Jia et al. [12] revealed a strong correlation between available nutrient content in the rhizosphere of *C. sinensis* germplasm resources and bacterial community composition, confirming the key role of rhizosphere microorganisms in nutrient mobilization. Beyond nutrient supply. Chen et al. [13] elucidated the mechanism by which microorganisms enhance stress resistance in tea plants through upregulating antioxidant enzyme activities, and Shao et al. [14] demonstrated that nitrogen-cycling functional microorganisms in mountain tea plantations regulate nitrogen cycling through specific response mechanisms. Collectively, these studies indicate that the structural and functional plasticity of rhizosphere microbial communities constitutes an important basis for the adaptation of *Camellia* plants to complex environments. In contrast, *C. reticulata* has received little research attention. Its rhizosphere microbial communities, including their vertical distribution characteristics and environmental response mechanisms, remain completely unexplored.

Thus, to address the effects of soil microbial diversity and the environmental drivers on the growth of *C. reticulata*, we hypothesize that (1) the microbial community composition, diversity, and co-occurrence network topology differ significantly in different soil layers; and (2) the microbial community structure and diversity exhibit significant vertical variations along the soil profile, and these variations are primarily driven by key environmental factors. To test these hypotheses, soil profile samples were collected from two depths (0–30 cm and 30–60 cm) across six wild *C. reticulata* populations in Yunnan Province. Soil physicochemical properties and enzyme activities were measured, and the composition of bacterial and fungal communities was analyzed using high-throughput sequencing (Illumina (San Diego, CA, USA) MiSeq platform). By identifying the key environmental factors associated with rhizosphere microbial community assembly, this study provides a scientific basis for understanding rhizosphere microecological processes and optimizing cultivation management of *C. reticulata*.

## 2. Materials and Methods

### 2.1. Research Area

The study area encompasses Kunming City, Chuxiong Yi Autonomous Prefecture, Dali Bai Autonomous Prefecture, and Baoshan City in Yunnan, China. The regional elevation ranges from 1951 to 2489 m above sea level, with mean annual temperatures between 15.0 and 20.0 °C and annual precipitation ranging from 800 to 1500 mm. The specific geographic coordinates and elevation data of each sampling site are detailed in Table 1.

### 2.2. Collection and Analysis of Samples

The sampling sites were all wild habitats without human management interventions, with consistent red soil across all locations. In October 2024, rhizosphere soil samples were collected from the distribution sites of *C. reticulata* listed in Table 1. Within each sampling site, soil samples were collected from two soil layers (0–30 cm and 30–60 cm) using an S-shaped multi-point sampling method with a soil auger (3.5 cm inner diameter). Multiple subsamples from the same plot and the same soil layer were thoroughly mixed to form a composite soil sample. Three independent biological replicates were established per sampling site, and a total of 36 soil samples were collected. The specific procedure was as follows: soil blocks containing intact root systems (representing the main root distribution zone) were excavated and gently shaken to remove loosely attached bulk soil. Subsequently, the soil firmly adhering to the root surfaces (0–5 mm from the root surface) was carefully brushed off and defined as rhizosphere soil. During the collection process, special care was taken to minimize damage to plant roots and to carefully remove any inadvertently included root fragments. To minimize exogenous contamination, the entire sampling and storage process was carried out with sterile tools. Personnel wore protective suits and sterile gloves, and direct hand contact with samples was strictly prohibited. Before each sampling, any residual material on the tool surfaces was wiped with sterile tissue. The collected composite soil samples were divided into two parts on site: one portion was placed in pre-labeled sterile resealable bags for physicochemical analysis, while the other was transferred into sterile centrifuge tubes, flash-frozen in liquid nitrogen, and subsequently stored at −80 °C to suppress microbial metabolic activity and chemical reactions, thereby preserving the original state of the samples as much as possible. Air-dried samples were ground and sieved through a 20-mesh sieve for the determination of soil physicochemical properties, while frozen samples were reserved for subsequent high-throughput sequencing analysis of soil microbial community structure [15].

### 2.3. Soil Physicochemical Properties and Enzyme Activities

Soil physicochemical parameters were determined following the methods described by Bao et al. [16]. Soil pH was measured using a pH meter with a soil-to-water ratio of 1:10 (mass basis). Soil organic matter (SOM) content was determined by the potassium dichromate-sulfuric acid (K_2_Cr_2_O_7_-H_2_SO_4_) oxidation method, with results expressed as g·kg^−1^. Total nitrogen (TN) was quantified using the Kjeldahl nitrogen determination method, with results expressed as g·kg^−1^. Total phosphorus (TP) was measured using the sodium oxide melting Jinkang colorimetric method, with results expressed as g·kg^−1^. Total potassium (TK) was determined via the sodium oxide melting flame photometric method, with results expressed as g·kg^−1^. Alkaline nitrogen (AN) was determined using the alkaline diffusion method, with results expressed as mg·kg^−1^. Available potassium (AK) was measured using the acetic acid extraction flame photometric method, with results expressed as mg·kg^−1^. Soil available phosphorus (AP) content was determined through the hydrochloric acid ammonium chloride extraction method, with results expressed as mg·kg^−1^.

Soil enzyme activities were assessed using chromogenic methods: phosphatase activity was determined by p-nitrophenyl phosphate colorimetry, urease activity by sodium phenolate-sodium hypochlorite colorimetry, and sucrase activity by 3,5-dinitrosalicylic acid colorimetry [17,18].

### 2.4. DNA Extraction, PCR Amplification, and Sequencing of Rhizosphere Soil

Total microbial community DNA was extracted from soil samples using the CretMag™ Power Soil DNA Kit (Cretaceous Biotechnology Co., Ltd., Suzhou, China) following the manufacturer’s instructions. After extraction, DNA integrity was verified by 1% agarose gel electrophoresis, and concentration and purity (A260/A280 ratio) were determined using a NanoDrop 2000 spectrophotometer (Thermo Fisher Scientific Inc., Waltham, MA, USA). For bacterial community analysis, the V3–V4 variable region of the 16S rRNA gene was amplified using primers 341F (5′-CCTAYGGGRBGCASCAG-3′) and 806R (5′-GGACTACHVGGGTWTCTAAT-3′). For fungal community analysis, the internal transcribed spacer (ITS) region of ribosomal RNA genes was amplified using primers ITS1F (5′-CTTGGTCATTTAGAGGAAGTAA-3′) and ITS2R (5′-GCTGCGTTCTTCATCGATGC-3′). PCR amplification was performed on an A200 PCR thermocycler (Hangzhou LongGene, Hangzhou, China) with the following program: initial denaturation at 94 °C for 2 min; 30 cycles of denaturation at 94 °C for 30 s, annealing at 55 °C for 30 s, and extension at 72 °C for 30 s; followed by a final extension at 72 °C for 10 min, and hold at 4 °C. Each 50 μL PCR reaction contained 25 μL of 2 × ES Taq MasterMix (Dye), 2 μL each of forward and reverse primers (10 μM), template DNA (10 ng), and ddH_2_O to final volume. Three technical replicates were performed per sample to ensure amplification reliability.

PCR products from the same sample were then pooled, separated by 2% agarose gel electrophoresis, and purified using the AxyPrep DNA Gel Extraction Kit (Axygen Biosciences, Union City, CA, USA). The purified products were examined by 2% agarose gel electrophoresis and quantified using a Quantus™ Fluorometer (Promega Corporation, Madison, WI, USA). Libraries were constructed using the VAHTS^®^ Universal Plus DNA Library Prep Kit for MGI V2 (Vazyme Biotech Co., Ltd., Nanjing, China) and sequenced on the DNBSEQ-G99 PE300 platform (BGI Group, Shenzhen, China) with paired-end mode. Raw sequencing reads were quality-filtered using fastp v0.20.0 (https://github.com/OpenGene/fastp, accessed on 15 February 2025) and merged using FLASH v1.2.7 (http://www.cbcb.umd.edu/, accessed on 15 February 2025). Operational taxonomic units (OTUs) were clustered at 97% similarity threshold using UPARSE v7.1 (http://drive5.com/uparse/, accessed on 15 February 2025), and chimeric sequences were removed during the clustering process. Taxonomic assignment of representative sequences was performed using the RDP classifier v2.14 (https://sourceforge.net/projects/rdp-classifier/, accessed on 16 February 2025) against the Silva database (https://www.arb-silva.de/, accessed on 16 February 2025) for bacterial 16S rRNA genes and the UNITE database (https://unite.ut.ee/, accessed on 16 February 2025) for fungal ITS sequences, with a confidence threshold of 70%.

### 2.5. Statistical Analysis

Statistical analysis was performed using SPSS 22.0 software. One-way analysis of variance (ANOVA) followed by Duncan’s multiple range test was used to determine the significance of differences (*p* < 0.05). Microbial community abundance plots were generated using Origin 2021 software. Based on the optimized sequence data, alpha diversity indices including Shannon, Simpson, and Chao1 were calculated using an online analysis platform (http://www.pronetscloud.com, accessed on 5 January 2026). Paired-sample *t*-tests were then performed to compare the differences in alpha diversity indices and the relative abundances of dominant microbial taxa between the surface (0–30 cm) and subsurface (30–60 cm) soil layers. Non-metric multidimensional scaling (NMDS) based on Bray–Curtis distances was also performed using the same platform to evaluate differences in microbial community structure among samples.

Data analysis was conducted using R software (version 4.30): Mantel test was performed to analyze correlations between key microbial communities and soil environmental factors [19]; Redundancy analysis (RDA) was used to explore the effects of environmental factors on microbial communities, with environmental factors standardized (z-score transformation) prior to analysis. Variance inflation factor (VIF) was employed to diagnose multicollinearity, with redundant variables (VIF > 10) being excluded [20]. To investigate the coexistence patterns among microbial taxa, this study constructed co-occurrence networks based on Spearman’s correlation coefficients. For each soil layer, phyla with an average relative abundance >1% and OTUs with an average relative abundance >0.01% were selected for correlation analysis. A stringent threshold (adjusted *p* < 0.05, |r| > 0.6) was applied to ensure the reliability of the associations. The networks were constructed based on a modified random matrix theory approach. Topological indices, including average connectivity, average clustering coefficient, and average path distance, were calculated using the igraph package to quantify network characteristics. Based on within-module connectivity (*Zi*) and among-module connectivity (*Pi*), nodes were classified into four functional categories: peripheral nodes (*Zi* < 2.5 and *Pi* < 0.62), module hubs (*Zi* ≥ 2.5 and *Pi* < 0.62), connectors (*Zi* < 2.5 and *Pi* ≥ 0.62), and network hubs (*Zi* ≥ 2.5 and *Pi* ≥ 0.62). Network graphs were visualized using Gephi (version 10.60), and *Zi*-*Pi* plots were generated using GraphPad Prism 10 to further analyze the topological features [21].

## 3. Results and Analysis

### 3.1. Soil Physicochemical Properties and Soil Enzyme Activities

The physicochemical properties of soil across different sampling sites and depth layers of *C. reticulata* are presented in Table 2. Soil pH in ZWY ranged from 6.10 to 6.22, indicating a near-neutral condition, while other sampling sites exhibited acidic soil with pH increasing with depth. Organic matter showed significant differences (*p* < 0.05) between ZSZ and YB in both 0–30 cm and 30–60 cm layers. Total nitrogen differed significantly (*p* < 0.05) only between ZSZ and YB in the 0–30 cm layer, and between MHC and YB in the 30–60 cm layer. Additionally, significant differences (*p* < 0.05) were observed across depths at site YB. Total phosphorus varied significantly (*p* < 0.05) only between ZWY and SDS in the 0–30 cm layer. Total potassium showed significant differences (*p* < 0.05) between the 0–30 cm and 30–60 cm layers. Alkali-hydrolyzable nitrogen exhibited significant differences (*p* < 0.05) among ZWY, ZSZ, and YB in the 0–30 cm layer, and between ZWY and YB in the 30–60 cm layer, with significant differences across depths observed at all sampling sites except SDS and HMC. Available phosphorus showed significant differences (*p* < 0.05) among all sampling sites in both 0–30 cm and 30–60 cm layers, as well as across depths in all sites except ZSZ and HMC. Available potassium exhibited significant differences (*p* < 0.05) among different sampling sites and soil layers.

Soil enzyme activities at different soil depths and sampling sites of *C*. *reticulata* are shown in Table 3. Urease activity in the 0–30 cm layer showed no significant difference between ZWY, ZSZ, and SDS, while ZWY and ZSZ differed significantly from HMC (*p* < 0.05). MHC and YB also exhibited significant differences (*p* < 0.05). In the 30–60 cm layer, ZSZ, HMC, and YB displayed significant differences (*p* < 0.05), with ZSZ and YB showing significant variations between different depths (*p* < 0.05). Acid phosphatase activity differed significantly among all sampling sites in the 0–30 cm layer (*p* < 0.05). In the 30–60 cm layer, MHC differed significantly from other sites (*p* < 0.05), and ZWY, SDS, and MHC showed significant variations between depths (*p* < 0.05). Sucrase activity exhibited significant differences between ZWY and ZSZ in the 0–30 cm layer (*p* < 0.05). In the 30–60 cm layer, ZWY and YB differed significantly from other sampling sites (*p* < 0.05), while ZSZ and MHC showed significant variations between depths (*p* < 0.05). Catalase activity in both the 0–30 cm and 30–60 cm layers showed significant differences between SDS and other sampling sites (*p* < 0.05).

### 3.2. Soil Microbial Composition and Diversity

**Figure 1:**
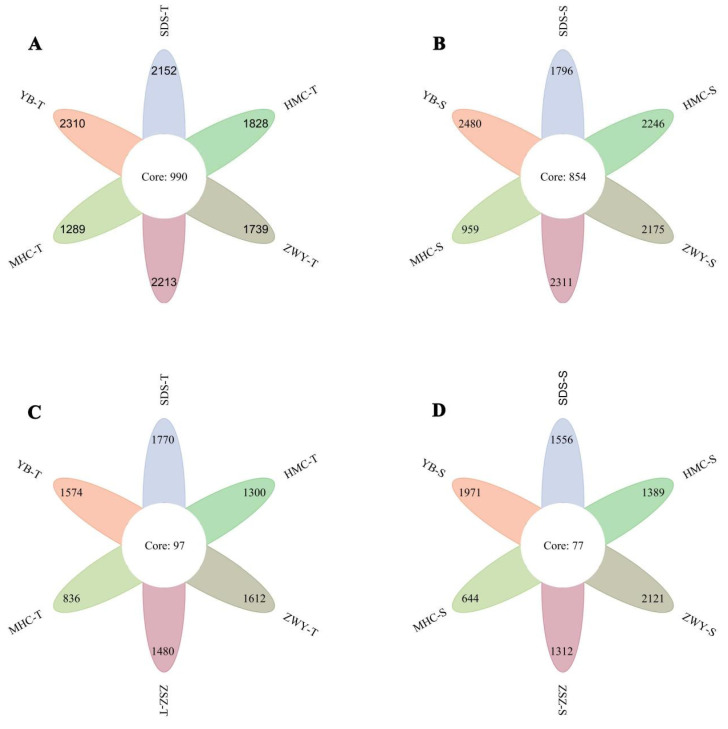
Venn diagrams showing shared and unique OTUs of bacterial (**A**,**B**) and fungal (**C**,**D**) communities at 0–30 cm (**A**,**C**) and 30–60 cm (**B**,**D**) soil depths. Numbers indicate OTUs shared across all samples within each depth.

The Venn diagram (Figure 1) shows that the number of bacterial operational taxonomic units (OTUs) shared across all samples in the 0–30 cm soil layer was 990, while in the 30–60 cm soil layer, the number of shared bacterial OTUs was 854. For fungi, the number of OTUs shared across all samples was 97 in the 0–30 cm layer and 77 in the 30–60 cm layer.

**Figure 2:**
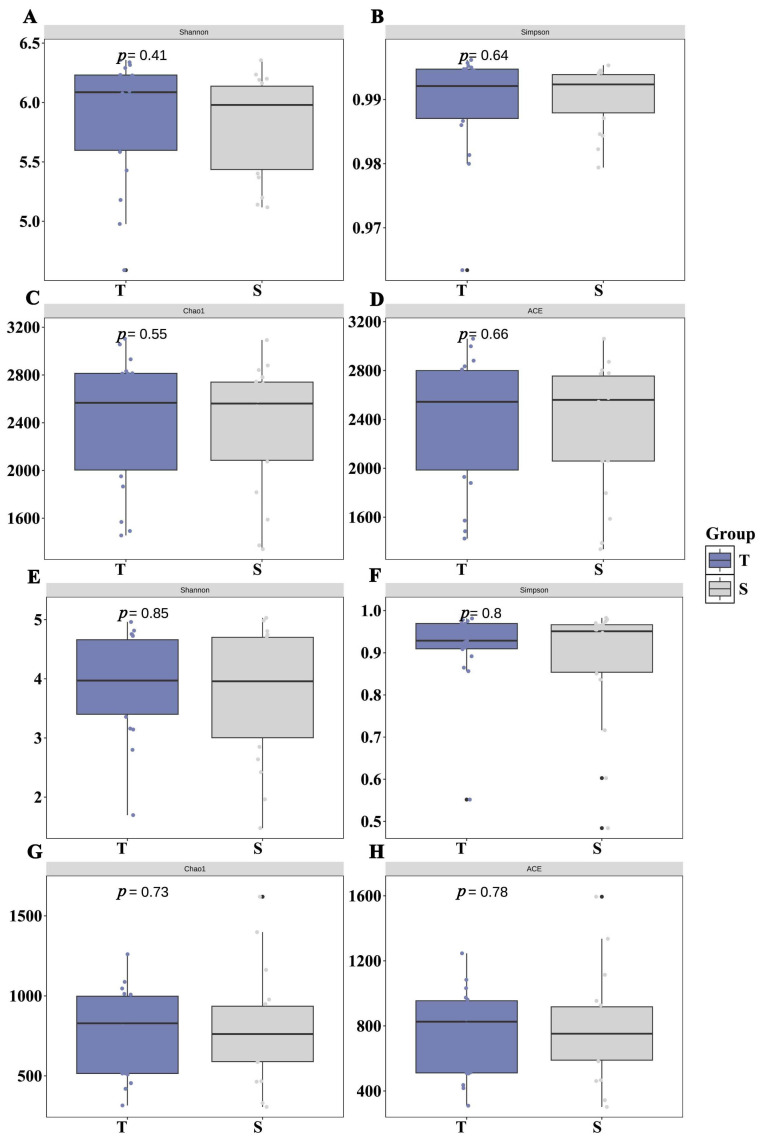
Comparison of bacterial (**A**–**D**) and fungal (**E**–**H**) alpha diversity indices between 0–30 cm (T) and 30–60 cm (S) soil depths. No significant differences were detected between depths for any index (independent samples t-test, *p* > 0.05, *n* = 18 per depth).

Alpha diversity analysis showed that the Ace, Chao, Shannon, and Simpson indices of both bacterial and fungal communities were generally higher in the 0–30 cm layer than in the 30–60 cm layer. Independent samples *t*-tests (*n* = 18 per depth) revealed that these differences were not statistically significant (*p* > 0.05 for all indices) (Figure 2).

**Figure 3:**
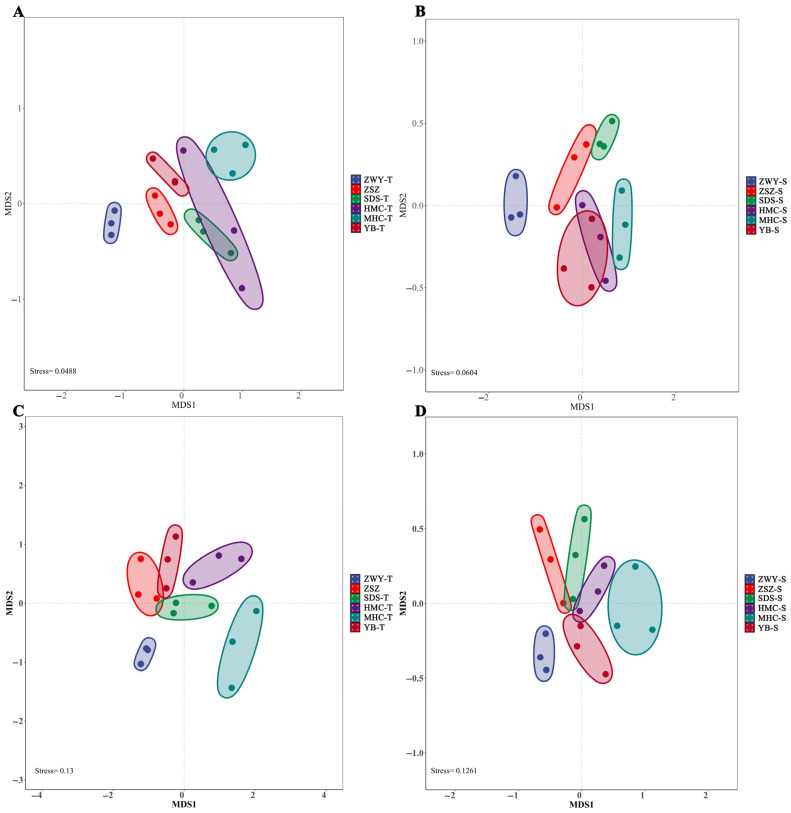
NMDS ordination of bacterial (**A**,**B**) and fungal (**C**,**D**) communities at 0–30 cm (**A**,**C**) and 30–60 cm (**B**,**D**) soil depths based on Bray–Curtis distances.

Based on the Bray–Curtis distance algorithm, non-metric multidimensional scaling (NMDS) was used to evaluate differences in diversity reflected by OTU abundance across sampling sites and soil depths (Figure 3). For bacterial communities, the HMC sample site was relatively distant from other sampling sites at both soil depths (0–30 cm and 30–60 cm). For fungal communities, the MHC sample site was distinctly separated from other sites in the 0–30 cm soil layer.

### 3.3. Compositional Characteristics of Rhizosphere Soil Microbial Communities at Phylum and Genus Levels

**Figure 4:**
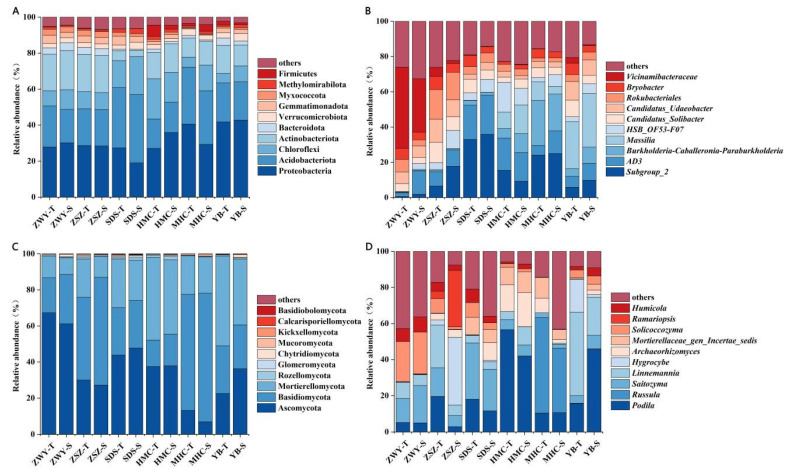
Relative abundance of soil bacteria (**A**,**B**) and fungi (**C**,**D**) at phylum (**A**,**C**) and genus (**B**,**D**) levels across sampling sites.

The analysis of the relative abundance of bacterial phyla and genera in the rhizosphere soil of *C. reticulata* across different habitats (Figure 4). The dominant bacterial and fungal groups were largely similar across different sampling sites, though slight variations were observed between sites and between soil depths within the same site.

At the bacterial phylum level (Figure 4A), the dominant groups were Proteobacteria, Acidobacteria, Chloroflexi, Actinobacteria, and Bacteroidota, in descending order, the result aligns with the typical distribution patterns of bacterial communities in most forest soils and also indicates that these phyla are core groups maintaining the rhizosphere microecological functions of *C. reticulata*. At the bacterial genus level (Figure 4B), the dominant genera were *Subgroup-2*, *AD-3*, *Burkholderia-Caballeronia-Paraburkholderia*, and *Massilia*. The relative abundance of *Vicinamibacteraceae* was higher in the two soil layers at the ZWY site compared to other sites.

Analysis of the relative abundance of bacterial phyla and genera in rhizosphere soils of *C. reticulata* across different habitats. At the fungal phylum level (Figure 4C), the dominant groups were Ascomycota, Basidiomycota, and Mortierellomycota in descending order, constituting the core of the rhizosphere fungal community of *C. reticulata*. These three phyla collectively dominated the ecological functions of the fungal community, the dominant genera were *Podila*, *Russula*, *Saitozyma*, and *Linnemannia*. The relative abundance of *Solicoccozyma* was higher in both soil layers at the ZWY site compared to other genera.

### 3.4. Correlation Analysis Between Soil Environmental Factors and Soil Microbial Communities in C. reticulata

**Figure 5:**
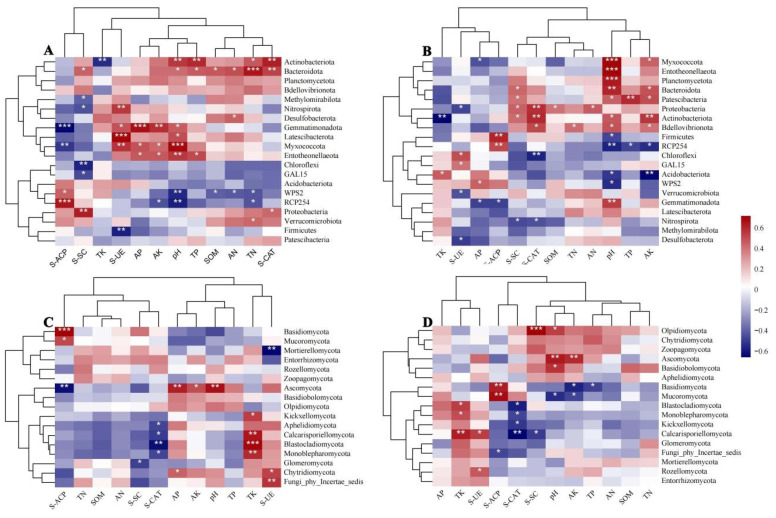
Spearman correlation heatmap showing relationships between soil environmental factors and microbial communities at different soil depths. (**A**) Bacteria at 0–30 cm; (**B**) Bacteria at 30–60 cm; (**C**) Fungi at 0–30 cm; (**D**) Fungi at 30–60 cm. Red and blue indicate positive and negative correlations, respectively. * *p* < 0.05, ** *p* < 0.01, *** *p* < 0.001.

As shown in the heatmap (Figure 5), the analysis of bacterial and fungal communities in the rhizosphere soil of *C. reticulata* revealed significant correlations with environmental variables. In the 0–30 cm soil layer (Figure 5A), pH was significantly positively correlated with Actinobacteriota, Bacteroidota, Gemmatimonadota, Latescibacterota, Myxococcota, and Entotheonellaeota. SOM was significantly positively correlated only with Bacteroidota. TN and TP were significantly positively correlated with Actinobacteriota and Bacteroidota. AN was significantly positively correlated with Bacteroidota and Desulfobacterota. AP and AK were significantly positively correlated with Gemmatimonadota, Myxococcota, and Entotheonellaeota. S-UE was significantly positively correlated with Nitrospirota, Gemmatimonadota, Latescibacterota, and Myxococcota. S-ACP was significantly positively correlated with WPS-2 and RCP254. S-SC was significantly positively correlated with Bacteroidota and Proteobacteria. S-CAT was significantly positively correlated with Actinobacteriota, Bacteroidota, and Proteobacteria. In the 30–60 cm soil layer (Figure 5B), pH was significantly positively correlated with Myxococcota, Entotheonellaeota, Planctomycetota, Bacteroidota, Patescibacteria, Actinobacteriota, and Gemmatimonadota. SOM was significantly positively correlated only with Proteobacteria. TN and AN were also significantly positively correlated with Proteobacteria. TP was significantly positively correlated with Patescibacteria, while TK was significantly positively correlated with Acidobacteriota. AP was significantly positively correlated with WPS-2. AK was significantly positively correlated with Myxococcota, Bacteroidota, Patescibacteria, Actinobacteriota, and Bdellovibrionota. S-UE was significantly positively correlated with Chloroflexi and GAL15. S-ACP was significantly positively correlated with Firmicutes and RCP254. Additionally, S-SC was significantly positively correlated with Bacteroidota, Patescibacteria, Proteobacteria, and Actinobacteriota, while S-CAT was significantly positively correlated with Proteobacteria, Actinobacteriota, and Bdellovibrionota.

In the 0–30 cm soil layer (Figure 5C), pH, available phosphorus (AP), and available potassium (AK) were all significantly positively correlated with Ascomycota. Total potassium (TK) was significantly positively correlated with Kickxellomycota, Calcarisporiellomycota, Blastocladiomycota, and Monoblepharomycota. Sucrase activity (S-UE) was significantly positively correlated with Chytridiomycota and Fungi_phy_Incertae_sedis, while acid phosphatase activity (S-ACP) was significantly positively correlated with Basidiomycota and Mucoromycota. In the 30–60 cm soil layer (Figure 5D), pH was significantly positively correlated with Olpidiomycota, Ascomycota, and Basidiobolomycota. TK was significantly positively correlated with Blastocladiomycota, Monoblepharomycota, and Calcarisporiellomycota. AK was significantly positively correlated with Ascomycota. Additionally, S-UE was significantly positively correlated with Calcarisporiellomycota and Rozellomycota, while S-ACP was also significantly positively correlated with Basidiomycota and Mucoromycota.

### 3.5. Soil Environmental Factors Influencing the Microbial Community Structure in the Rhizosphere of C. reticulata

To investigate the effects of soil physicochemical factors and enzyme activities on microbial community structure, redundancy analysis (RDA) was employed to further elucidate their relationships. The RDA results indicated that the first two ordination axes (RDA1 and RDA2) effectively reflected the extent to which environmental factors explained the variation in microbial communities.

**Figure 6:**
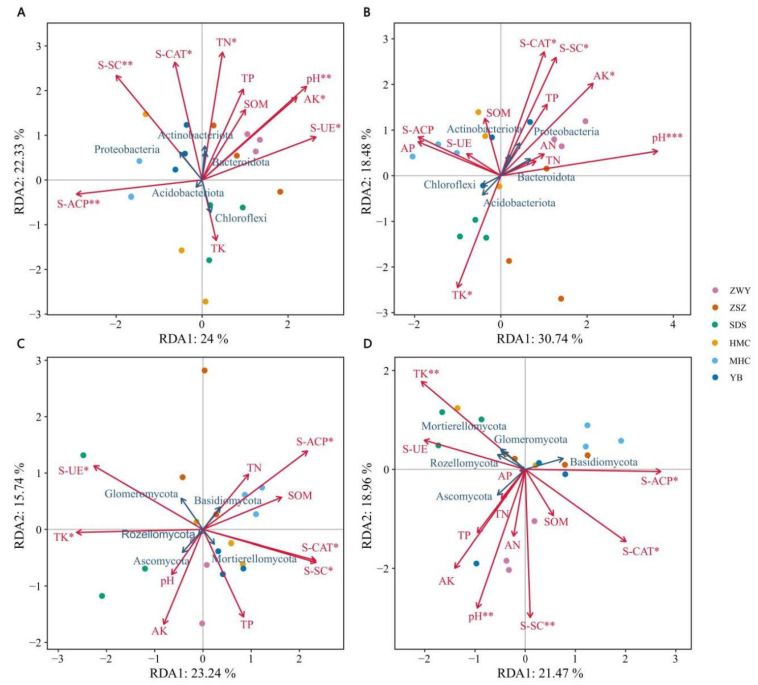
Redundancy analysis (RDA) of soil microbial communities (at the phylum level) and soil environmental factors. (**A**) Bacterial community at phylum level (0–30 cm soil depth); (**B**) Bacterial community at phylum level (30–60 cm soil depth); (**C**) Fungal community at phylum level (0–30 cm soil depth); (**D**) Fungal community at phylum level (30–60 cm soil depth). * *p* < 0.05, ** *p* < 0.01, *** *p* < 0.001. Red arrows represent quantitative environmental parameters, while blue arrows represent species. The length of the arrows indicates the magnitude of the influence of environmental factors on species data. Positive correlations are represented by acute angles, negative correlations by obtuse angles, and right angles indicate no correlation. The relative influence of environmental factors on the distribution of community composition across sampling sites is represented by the projection distance from sampling site points to the origin along the direction of the quantitative environmental factor arrow.

The first two axes explained 24.00% and 22.33% of the total variation in the bacterial community of the 0–30 cm rhizosphere soil (Figure 6A), cumulatively accounting for 46.33%. In the 30–60 cm layer, the first two axes explained 30.74% and 18.48%, cumulatively accounting for 49.22% (Figure 6B). In the 0–30 cm soil layer, sucrase activity (S-SC), catalase activity (S-CAT), total nitrogen (TN), pH, available potassium (AK), and urease activity (S-UE) were significantly positively correlated with Proteobacteria, Actinobacteriota, and Bacteroidota. In contrast, acid phosphatase activity (S-ACP) was significantly positively correlated with Acidobacteriota. Additionally, S-SC, S-CAT, and TN were significantly negatively correlated with Chloroflexi, while pH, AK, and S-UE showed significant negative correlations with Acidobacteriota. In the 30–60 cm soil layer, S-CAT, S-SC, AK, and pH were similarly significantly positively correlated with Actinobacteriota, Proteobacteria, and Bacteroidota, whereas total potassium (TK) was significantly positively correlated with Chloroflexi and Acidobacteriota. Conversely, S-CAT, S-SC, AK, and pH were significantly negatively correlated with Chloroflexi and Acidobacteriota, while TK showed a significant negative correlation with Actinobacteriota, Proteobacteria, and Bacteroidota.

In the 0–30 cm soil layer, the first two RDA axes explained 23.24% and 15.74% of the variation in the fungal community, cumulatively accounting for 38.98% (Figure 6C). In the 30–60 cm layer, the first two axes explained 21.47% and 18.96%, cumulatively accounting for 40.43% (Figure 6D). In the 0–30 cm soil layer, S-UE and TK were significantly positively correlated with Glomeromycota, Rozellomycota, and Ascomycota, while acid phosphatase activity (S-ACP), catalase activity (S-CAT), and sucrase activity (S-SC) were significantly positively correlated with Basidiomycota and Mortierellomycota. Conversely, S-UE and TK were significantly negatively correlated with Basidiomycota and Mortierellomycota, whereas S-ACP, S-CAT, and S-SC showed significant negative correlations with Glomeromycota, Rozellomycota, and Ascomycota. In the 30–60 cm soil layer, TK was significantly positively correlated with Mortierellomycota, Rozellomycota, and Glomeromycota; pH and S-SC were significantly positively correlated with Ascomycota; and S-ACP and S-CAT were significantly positively correlated with Basidiomycota. TK exhibited a significant negative correlation with Basidiomycota; pH and S-SC were significantly negatively correlated with Mortierellomycota, Rozellomycota, Glomeromycota, and Basidiomycota; while S-ACP and S-CAT showed significant negative correlations with Mortierellomycota, Rozellomycota, Glomeromycota, and Ascomycota.

**Figure 7:**
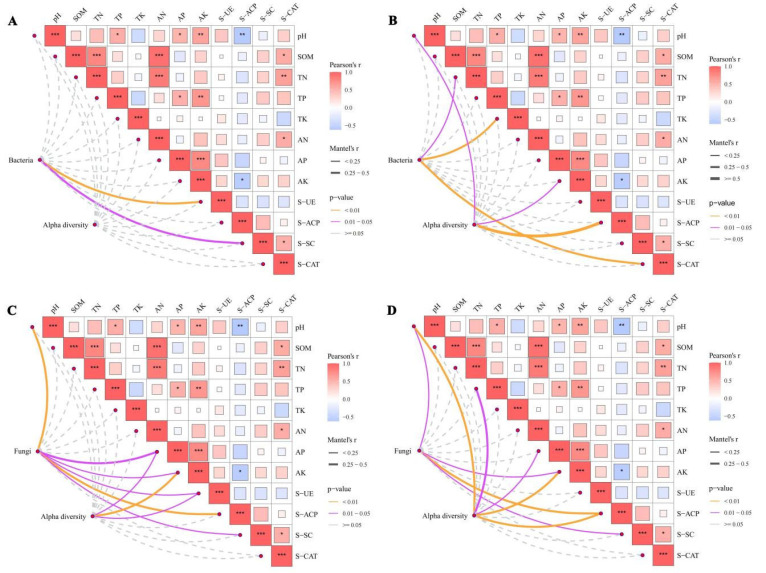
Partial Mantel tests based on Bray–Curtis distances were performed to analyze the correlations between soil microbial communities and environmental factors, with results visualized as color gradients based on Spearman’s correlation coefficients. The results for different soil layers and microbial taxa are shown in (**A**) (0–30 cm bacteria), (**B**) (30–60 cm bacteria), (**C**) (0–30 cm fungi), and (**D**) (30–60 cm fungi), respectively. * *p* < 0.05, ** *p* < 0.01, *** *p* < 0.001.

Mantel test analysis of soil microbial communities (at the phylum level) with enzyme activities and soil physicochemical properties revealed that (Figure 7): pH exhibited significant positive correlations with TP, AP, and AK; SOM showed significant positive correlations with TN, AN, and S-CAT; TN was significantly positively correlated with AN; TP demonstrated significant positive correlations with AP and AK; AN was significantly positively correlated with S-CAT; and AP showed a significant positive correlation with AK. Additionally, S-SC was significantly positively correlated with S-CAT.

Regarding bacterial communities (Figure 7A,B): At 0–30 cm soil depth, bacterial community abundance was significantly positively correlated with S-UE and S-SC (Figure 7A). At 30–60 cm soil depth, bacterial community abundance exhibited significant positive correlations with SOM, TP, and S-CAT, while bacterial community diversity was significantly positively correlated with pH and S-ACP (Figure 7B).

Regarding fungal communities (Figure 7C,D): At 0–30 cm soil depth, fungal community abundance was significantly positively correlated with pH, AP, AK, S-UE, S-ACP, and S-SC, whereas fungal community diversity showed significant positive correlations with AP, AK, and S-UE (Figure 7C). At 30–60 cm soil depth, fungal community abundance was significantly positively correlated with pH, AK, S-ACP, and S-SC, while fungal community diversity was significantly positively correlated with pH, TN, AP, AK, and S-ACP (Figure 7D).

### 3.6. Network Analysis of Fungal and Bacterial Communities in Different Habitats

**Figure 8:**
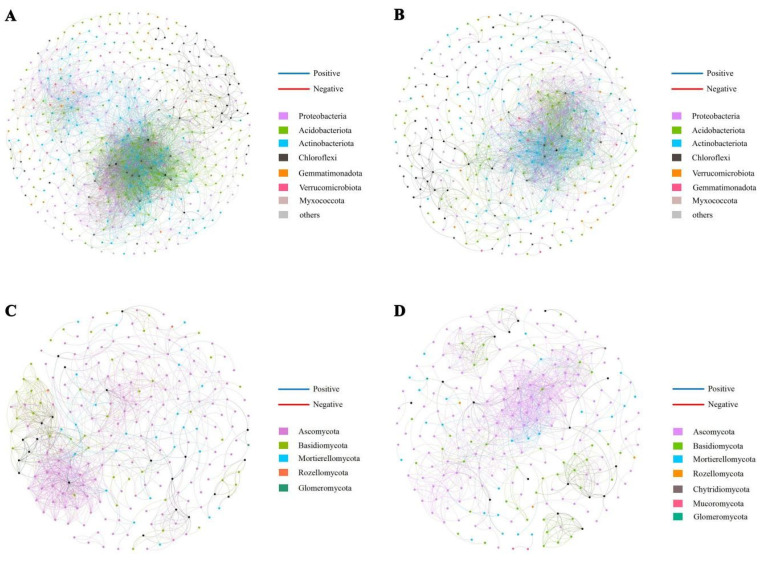
Co-occurrence networks of soil bacterial (**A**,**B**) and fungal (**C**,**D**) communities at 0–30 cm (**A**,**C**) and 30–60 cm (**B**,**D**) soil depths across different habitats. Node size represents relative abundance and connectivity (number of connections); red and blue lines indicate positive and negative correlations, respectively.

To elucidate the interaction patterns and ecological niche-sharing characteristics of keystone microorganisms in the rhizosphere soil of *C. reticulata* across different sampling sites and soil depths, microbial co-occurrence networks were constructed (Figure 8). The topological structures of bacterial and fungal networks exhibited significant differences between the 0–30 cm and 30–60 cm soil layers. In terms of network associations (Table 4), network analysis of both soil layers revealed that connectivity, number of edges, average degree, clustering coefficient, and path length of bacterial networks markedly decreased with increasing soil depth. In contrast, fungal network connectivity, number of edges, average degree, clustering coefficient, and path length showed a slight increasing trend with soil depth.

In the 0–30 cm soil layer, the bacterial network comprised 3823 positive correlations and 824 negative correlations, whereas in the 30–60 cm soil layer, the numbers of positive and negative correlations were 2152 and 233, respectively. For the fungal network, the 0–30 cm soil layer exhibited 1013 positive correlations and 25 negative correlations, while the 30–60 cm soil layer showed 1056 positive correlations and 13 negative correlations.

**Figure 9:**
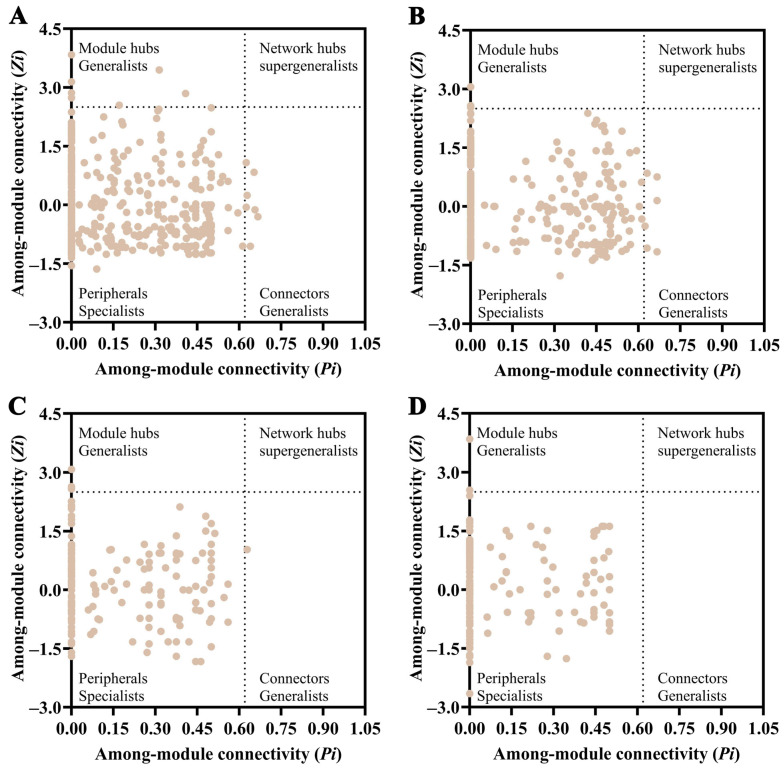
*Zi-Pi* plot analysis of topological features of soil bacterial and fungal communities across different soil depths. (**A**) Bacterial community at 0–30 cm soil depth; (**B**) Bacterial community at 30–60 cm soil depth; (**C**) Fungal community at 0–30 cm soil depth; (**D**) Fungal community at 30–60 cm soil depth. Each symbol represents an individual OTU. The horizontal dashed line indicates the threshold for within-module connectivity (*Zi* = 2.5), and the vertical dashed line indicates the threshold for among-module connectivity (*Pi* = 0.62). Nodes are classified into four categories: peripheral nodes (*Zi* < 2.5, *Pi* < 0.62), module hubs (*Zi* ≥ 2.5, *Pi* < 0.62), connectors (*Zi* < 2.5, *Pi* ≥ 0.62), and network hubs (*Zi* ≥ 2.5, *Pi* ≥ 0.62).

According to the *Zi-Pi* plot based on network topological analysis (Table 5 and Table 6; Figure 9). In the bacterial co-occurrence network of the 0–30 cm soil layer, seven module hubs were identified, predominantly belonging to the following phyla: Acidobacteriota (4 OTUs), Proteobacteria (2 OTUs), and Actinobacteriota (1 OTU). In the 30–60 cm soil layer network, two module hubs were identified, both assigned to Chloroflexi (2 OTUs). The compositional differences in keystone taxa between soil layers may reflect the influence of resource availability on microbial niche differentiation: resource-rich surface soils support multiple phyla cooperating to serve as network backbones, while in resource-limited deeper soils, specific phyla such as Chloroflexi assume core network functions.

Regarding connectors, the 0–30 cm soil layer network contained seven keystone connector nodes, taxonomically classified as follows: Acidobacteriota (3 OTUs), Chloroflexi (2 OTUs), Proteobacteria (1 OTU), and Actinobacteriota (1 OTU). In the 30–60 cm soil layer network, six connector nodes were identified, belonging to Actinobacteriota (3 OTUs), Acidobacteriota (2 OTUs), and Proteobacteria (1 OTU), respectively. In the fungal co-occurrence network, only three module hubs and one connector were identified in the 0–30 cm soil layer, all assigned to Ascomycota. In the 30–60 cm soil layer network, two module hubs were identified, also entirely belonging to Ascomycota. Ascomycota, as the sole keystone phylum in the fungal networks, underscores its core ecological role in organic matter decomposition and nutrient mineralization.

## 4. Discussion

### 4.1. Variations in Soil Microbial Community Composition Along the Soil Profile Depth

Soil microbial communities are key components of soil ecosystems, primarily composed of bacteria and fungi [22]. They drive essential ecological processes such as organic matter decomposition, nutrient cycling, and gas fluxes, while regulating soil environmental factors that indirectly influence plant nutrient uptake and stress resistance [23]. Although plants can fix carbon through photosynthesis, the nutrients required for plant growth are predominantly derived from microbial-mediated transformation and cycling processes in the soil, which directly determine the relationship between soil and plant growth [24].

In this study, the alpha diversity of both bacterial and fungal communities exhibited a decreasing trend with increasing soil depth (Figure 2), which is consistent with patterns commonly observed in forest [25] and agricultural [26] ecosystems. This vertical decline can be primarily attributed to the following ecological mechanisms: First, with increasing soil depth, the input of plant litter and root exudates decreases, leading to a significant reduction in carbon source availability, which limits the support for copiotrophic microbial taxa (e.g., Proteobacteria and Actinobacteriota) [27,28]. Second, nutrient limitation intensifies, as indicated by the decline in available nitrogen and phosphorus contents in deeper soil layers (Table 1), which selects for oligotrophic microorganisms adapted to nutrient-poor environments (e.g., Acidobacteriota and Chloroflexi) [29]. Additionally, poor aeration and enhanced anaerobic conditions in deeper soil layers further shape the functional metabolic types of microbial communities [30,31].

At the community composition level, the dominant bacterial phyla in this study were Proteobacteria, Acidobacteriota, Chloroflexi, Actinobacteriota, and Bacteroidota, while the dominant fungal phyla were Ascomycota, Basidiomycota, and Mortierellomycota (Figure 4). This compositional pattern is similar to that of soil microbial communities associated with *C. sinensis* [32,33]. Different phyla exhibited distinct responses to soil depth: copiotrophic groups such as Proteobacteria and Actinobacteriota showed higher relative abundances in surface soils, whereas oligotrophic groups such as Acidobacteriota and Chloroflexi increased in deeper layers (Figure 4). This vertical differentiation pattern is consistent with observations by Lv et al. [34] in forest soils, supporting the theoretical hypothesis that “resource gradients drive microbial niche differentiation.” Notably, the enrichment of Acidobacteriota in deeper soils aligns with their ecological characteristics as “K-strategists”—microorganisms that grow slowly but efficiently utilize scarce resources and maintain survival under environmental stress [35].

### 4.2. Correlation Between Soil Microbial Communities and Soil Environmental Factors

Integrated analysis of Spearman’s correlation, RDA, and Mantel test results (Figure 5, Figure 6 and Figure 7) revealed that pH, potassium (K) content, and catalase activity (S-CAT) are the core environmental drivers regulating the assembly of rhizosphere microbial communities in *C. reticulata*. This finding is consistent with the results reported by Zong [36]. Total potassium (TK) was identified as the most important environmental factor influencing soil fungal communities across soil layers, which aligns with the findings of Luo [37] and Wan et al. [38].

Soil pH variation was found to exert a significant impact on microbial diversity [39]. Changes in pH may not only lead to denaturation and inactivation of key soil enzymes, thereby reducing nutrient solubility and plant availability, but also exacerbate soil acidification, promoting the dissolution of aluminum ions and certain heavy metals. This process enhances their direct phytotoxicity and suppresses soil microbial activity and diversity [40]. In the present study, soil pH exhibited an increasing trend with increasing rhizosphere soil depth (Table 1). This pattern may be attributed to two main factors: first, the application of nitrogen fertilizers such as urea and ammonium sulfate increases the concentration of exchangeable Al^3+^ and H^+^ in the soil, thereby intensifying soil acidification [41]; second, plant roots are more densely distributed in surface layers, where organic acids and alcohols secreted by roots accumulate in the plow layer, further lowering local pH [42].

Potassium (K) emerged as the most influential nutrient factor shaping the rhizosphere microbial community of *C. reticulata* in this study, and its role should not be underestimated. Soil potassium not only activates multiple enzyme systems and enhances nutrient transformation efficiency but also plays a critical role in regulating the rhizosphere microenvironment, facilitating the colonization of beneficial microorganisms, and reshaping the structure of rhizosphere microbial communities [43].

Regarding soil enzyme activities, catalase activity (S-CAT) was identified as a key factor influencing the rhizosphere soil of *C. reticulata*. In the study by Chen [44], catalase activity was also reported as a major environmental determinant affecting Proteobacteria. Collectively, soil pH, potassium, and S-CAT serve as core drivers co-regulating the assembly of rhizosphere microbial communities in *C. reticulata*.

### 4.3. Shifts in Soil Microbial Co-Occurrence Networks Along the Soil Depth Gradient

Microbial co-occurrence network analysis can reveal previously unrecognized patterns of microbial coexistence, thereby elucidating the complex relationships among different microbial taxa. In this study, we found that the average clustering coefficient of both fungal and bacterial networks decreased with increasing soil depth (Table 3), indicating that the network complexity of bacterial and fungal communities declined as soil depth increased. This suggests a higher degree of habitat heterogeneity in surface soils [45]. Similarly, the higher average clustering coefficient observed in surface soils indicates more connections within modules than between modules, implying that soil bacteria occupying similar ecological niches are more closely associated than those in different niches [46]. This study also found that the average path distance of fungal networks increased with soil depth, whereas that of bacterial networks decreased. Average path distance characterizes the tightness of connections between species (nodes) in a network; a smaller average path distance indicates closer interactions among species within the network. However, a more tightly connected community also implies faster propagation of external disturbances through the network, potentially making it more sensitive to perturbations [47]. This suggests that deep-layer fungal networks are more stable in response to disturbances, while the opposite is true for bacteria. Additionally, with increasing soil depth, the modularity of bacterial networks increased, while that of fungal networks decreased. Network modularity is commonly used to indicate the resistance of a system to external disturbances; higher modularity confers greater resistance to such disturbances [48]. Therefore, in the soils of different *C. reticulata* habitats, bacterial networks were more stable in deeper layers, whereas fungal networks exhibited greater stability in surface layers.

Based on *Zi-Pi* analysis, Acidobacteriota and Ascomycota were identified as highly connected taxa within the rhizosphere microbial networks of *C. reticulata* (Table 5 and Table 6). It should be noted that such “module hubs” and “connectors,” identified based on network topological features, represent nodes with high connectivity within ecological networks, suggesting their potential importance in maintaining network structure; however, they are not directly equivalent to ecologically defined “keystone functional taxa.” In this study, highly connected taxa within the bacterial network were identified across both soil layers. Among fungi, Ascomycota was the sole highly connected phylum within the fungal networks. These results highlight the importance of Acidobacteriota and Ascomycota in maintaining network structure along the deep soil profile in the restored ecosystem under investigation. Within the *C. reticulata* soil ecosystem, members of Acidobacteriota are considered to contribute to nutrient cycling and organic matter decomposition [49,50]. They are known for their tolerance to a wide range of soil pH and are particularly abundant in acidic soils. Some members of Acidobacteriota are classified as K-strategists, meaning they can thrive in nutrient-limited environments with slow growth rates and high tolerance to toxic compounds [35]. The majority of Ascomycota taxa are saprophytic fungi, driving carbon and nitrogen cycling in plant–soil systems through the degradation of substances such as cellulose, thereby playing an important role in soil nutrient mobilization [51]. As highly connected taxa identified within the bacterial and fungal networks, Acidobacteriota and Ascomycota may play important roles in maintaining the stability of the microbial network in the *C. reticulata* soil ecosystem. Their potential involvement in nutrient cycling and organic matter transformation could contribute to the ecological functioning of deep soil layers, though experimental validation is needed to confirm their specific functional roles.

## 5. Conclusions

This study investigated the vertical distribution patterns of rhizosphere soil microbial communities in *C. reticulata* across the 0–30 cm and 30–60 cm soil layers, revealing the responses of microbial community composition, diversity, and co-occurrence networks to soil depth gradients. Soil depth significantly influenced microbial community structure: the alpha diversity of both bacterial and fungal communities decreased with increasing soil depth, and community composition exhibited distinct vertical differentiation, with copiotrophic phyla (e.g., Proteobacteria and Actinobacteriota) showing higher relative abundances in surface soils, while oligotrophic phyla (e.g., Acidobacteriota and Chloroflexi) were enriched in deeper layers. Co-occurrence network analysis revealed that bacterial network complexity decreased with increasing soil depth, whereas fungal networks exhibited the opposite trend, suggesting divergent ecological strategies of bacteria and fungi in response to environmental gradients. Environmental factor analysis indicated that soil pH, potassium (K) content, and catalase activity (S-CAT) were significantly correlated with microbial community structure, representing major environmental factors influencing rhizosphere microbial assembly in *C. reticulata*. Network topological analysis further identified Acidobacteriota and Ascomycota as highly connected taxa within the microbial networks, suggesting their potential importance in maintaining network structure. This study provides fundamental data for understanding the vertical differentiation patterns of rhizosphere microbial communities in *C. reticulata* and their environmental response mechanisms, and offers a theoretical reference for the targeted regulation of rhizosphere microecology in *C. reticulata* cultivation management.

## Figures and Tables

**Table 1:** The list of the location of soil samples.

No.	Study Area	Longitude (E)	Latitude (N)	Elevation (m)
1	Kunming Botanical Garden (Chinese Academy of Sciences)	102°74′	25°14′	1951
2	Zhongshan Town, Chuxiong	101°10′	24°90′	2342
3	Shidong Temple, Fengqing	100°20′	24°27′	2073
4	Hemu Village, Tengchong	98°30′	25°10′	1967
5	Meihua Village, Yongping	99°38′	25°30′	2456
6	Yangbi Yi Autonomous County, Dali	99°51′	25°38′	2489

**Table 2:** Physicochemical properties of rhizosphere soil at different soil depths.

Soil Depth	Sampling Sites	pH	Soil Organic Matter	Total N	Total *p*	Total K	Alkali- Hydrolyzable N	Available *p*	Available K
0–30 cm	ZWY	6.10 ± 0.13 Aa	63.18 ± 10.98 Ab	4.93 ± 0.04 Ab	2.46 ± 0.46 Aa	7.58 ± 0.31 Ae	350.45 ± 36.57 Ac	60.24 ± 2.05 Aa	457.64 ± 35.15 Aa
	ZSZ	5.59 ± 0.48 Ab	114.91 ± 6.41 Aa	7.59 ± 1.94 Aa	1.29 ± 0.08 Abc	17.16 ± 3.50 Ac	623.00 ± 7.47 Aa	9.40 ± 1.70 Ade	220.79 ± 6.58 Ab
	SDS	5.30 ± 0.09 Abc	29.69 ± 11.24 Ac	2.64 ± 0.86 Ab	1.10 ± 0.08 Ac	29.05 ± 1.22 Aa	212.61 ± 54.29 Ad	45.18 ± 6.34 Ab	254.58 ± 30.43 Ab
	HMC	5.41 ± 0.09 Bb	60.43 ± 17.42 Ab	4.60 ± 2.20 Ab	1.31 ± 0.20 Abc	13.18 ± 1.04 Ad	231.20 ± 40.20 Ad	3.94 ± 1.94 Ae	100.29 ± 29.11 Bc
	MHC	4.97 ± 0.08 Ac	46.63 ± 8.32 Abc	2.91 ± 0.38 Ab	1.30 ± 0.44 Abc	12.27 ± 1.11 Ad	253.79 ± 12.21 Ad	14.61 ± 3.65 Bd	100.37 ± 27.84 Ac
	YB	5.41 ± 0.12 Ab	109.16 ± 9.67 Aa	7.49 ± 0.83 Aa	1.94 ± 0.69 Aab	21.69 ± 1.20 Ab	550.85 ± 38.58 Ab	32.38 ± 1.88 Ac	425.62 ± 12.95 Aa
30–60 cm	ZWY	6.22 ± 0.20 Aa	38.26 ± 24.30 Ab	2.82 ± 1.52 Aab	1.71 ± 0.64 Aa	8.82 ± 1.56 Ad	249.00 ± 2.00 Bc	15.26 ± 0.11 Bb	511.00 ± 11.00 Aa
	ZSZ	5.70 ± 0.18 Ab	38.18 ± 1.04 Bb	4.89 ± 2.20 Aab	1.09 ± 0.32 Aa	16.25 ± 3.69 Abc	293.33 ± 7.77 Bb	5.42 ± 3.24 Ac	166.30 ± 6.70 Bc
	SDS	5.40 ± 0.12 Ac	31.78 ± 6.40 Ab	2.83 ± 0.65 Aab	1.14 ± 0.24 Aa	26.27 ± 3.56 Aa	225.00 ± 5.56 Ad	20.78 ± 5.89 Bab	163.70 ± 3.20 Bc
	HMC	5.60 ± 0.08 Abc	43.31 ± 10.61 Aab	3.84 ± 1.13 Aab	1.47 ± 0.54 Aa	13.88 ± 3.14 Acd	289.33 ± 4.47 Ab	6.14 ± 4.53 Ac	500.33 ± 34.00 Aa
	MHC	5.09 ± 0.11 Ad	43.93 ± 4.12 Aab	2.63 ± 0.33 Ab	1.24 ± 0.48 Aa	12.35 ± 1.70 Acd	215.00 ± 10.44 Bd	26.19 ± 5.98 Aa	77.07 ± 22.44 Ad
	YB	5.59 ± 0.09 Abc	61.98 ± 1.31 Ba	5.20 ± 0.52 Ba	1.85 ± 0.55 Aa	19.65 ± 2.81 Ab	396.33 ± 25.79 Ba	20.25 ± 4.87 Bab	231.33 ± 3.00 Bb

Note: Data are presented as mean ± standard deviation. Different uppercase letters indicate significant differences among different soil depths at the same sampling site, while different lowercase letters indicate significant differences among different sampling sites at the same soil depth (*p* < 0.05).

**Table 3:** Enzyme activities in rhizosphere soil under different habitats.

Soil Depth	Sampling Sites	Urease	Phosphatase	Sucrase	Catalase
0–30 cm	ZWY	344.63 ± 26.57 Aa	15.42 ± 3.57 d	15.61 ± 3.15 Ab	23.83 ± 1.00 Aa
	ZSZ	359.23 ± 2.68 Aa	21.71 ± 0.20 Ab	12.58 ± 1.69 Abc	23.59 ± 1.52 Aa
	SDS	348.51 ± 9.74 Aa	16.67 ± 1.98 Bcd	9.80 ± 1.90 Ac	19.99 ± 0.29 Ab
	HMC	290.18 ± 24.37 Ab	20.35 ± 1.72 Abc	9.71 ± 3.59 Ac	22.94 ± 1.59 Aa
	MHC	307.10 ± 6.42 Ab	44.21 ± 2.22 Aa	23.03 ± 4.13 Aa	23.23 ± 1.63 Aa
	YB	298.73 ± 20.31 Ab	20.45 ± 3.93 Abc	26.48 ± 2.26 Aa	25.25 ± 1.16 Aa
30–60 cm	ZWY	321.80 ± 13.44 Aa	21.48 ± 0.74 b	22.98 ± 4.74 Aa	22.72 ± 0.62 Aa
	ZSZ	210.75 ± 5.63 Bc	21.60 ± 1.12 Ab	8.23 ± 2.06 Bb	21.67 ± 1.15 Aa
	SDS	345.15 ± 26.42 Aa	19.81 ± 3.95 Ab	8.76 ± 1.79 Ab	18.48 ± 2.17 Ab
	HMC	309.58 ± 47.86 Aa	18.23 ± 5.35 Ab	11.59 ± 5.64 Ab	22.24 ± 1.08 Aa
	MHC	291.81 ± 57.42 Aab	32.62 ± 7.98 Ba	13.45 ± 6.34 Bb	22.84 ± 1.77 Aa
	YB	232.90 ± 20.21 Bbc	21.98 ± 1.22 Ab	24.57 ± 3.02 Aa	23.07 ± 0.90 Aa

Note: Data are presented as mean ± standard deviation. Different uppercase letters indicate significant differences among different soil depths at the same sampling site, while different lowercase letters indicate significant differences among different sampling sites at the same soil depth (*p* < 0.05).

**Table 4:** Topological characteristics of soil bacterial and fungal co-occurrence networks in different habitats.

Network Indexes	Bacteria		Fungi	
	0–30 cm	30–60 cm	0–30 cm	30–60 cm
Node	517	395	247	248
Link	4647	2385	1038	1069
positive correlation	3823	2152	1013	1056
negative correlation	824	233	25	13
average clustering coefficient	0.549	0.511	0.619	0.595
Average path distance	3.782	3.778	4.226	4.408
Modularity	0.358	0.386	0.650	0.591

**Table 5:** Distribution of module hubs and connectors in soil bacterial networks across different soil depths.

Classification of Bacteria (Phylum)	Number of Modular Hub		Number of Connection Node	
	0–30 cm	30–60 cm	0–30 cm	30–60 cm
Proteobacteria	2	0	1	1
Acidobacteriota	4	0	3	2
Actinobacteriota	1	0	1	3
Chloroflexi	0	2	1	0

Values represent the number of OTUs identified as module hubs or connectors within each phylum.

**Table 6:** Distribution of module hubs and connectors in soil fungal networks across different soil depths.

Classification of Fungal (Phylum)	Number of Modular Hub		Number of Connection Node	
	0–30 cm	30–60 cm	0–30 cm	30–60 cm
Ascomycota	3	2	1	0

Values represent the number of OTUs identified as module hubs or connectors within each phylum.

## Data Availability

The original contributions presented in this study are included in the article. Further inquiries can be directed to the corresponding author.
